# The effect of HIV on patients’ lives: a phenomenological qualitative study

**DOI:** 10.1080/17482631.2024.2315634

**Published:** 2024-02-10

**Authors:** Naif S. Alzahrani, Abdulaziz Mofdy Almarwani

**Affiliations:** a Department of Medical Surgical Nursing, College of Nursing, Taibah University, Medina, Saudi Arabia; b Department of Psychiatric Nursing, College of Nursing, Taibah University, Medina, Saudi Arabia

**Keywords:** Human immunodeficiency virus (HIV), fear, hopelessness, qualitative phenomenological, Saudi Arabia

## Abstract

**Introduction:**

Human immunodeficiency virus (HIV) infection poses a significant threat to the immune system, compromising the body’s ability to combat diseases and infections. The Ministry of Health in Saudi Arabia reported an HIV incidence rate of 3 cases per 10,000 individuals. This study aimed to gain insight into the lived experience of Saudi patients living with HIV.

**Methods:**

Employing a qualitative phenomenological approach, this study conducted in-depth interviews with 16 HIV patients (10 men, 6 women) between January 2023 and May 2023.

**Results:**

Thematic data analysis highlighted three overarching themes and four subthemes. “Fear of the Future” encompassed subthemes including the fear of infecting a family member, fear of marriage, fear of employment recruitment, and fear of scandals. “Hopelessness” reflected the profound emotional state experienced by patients. “Overcoming Adversity” captured the resilience and strength demonstrated by individuals facing the challenges of living with HIV.

**Conclusion:**

Saudi patients diagnosed with HIV encounter numerous obstacles in their daily lives. The fear of the future, including concerns such as infecting family members, marriage prospects, employment opportunities, and potential social repercussions, significantly impacts their overall well-being. By understanding the lived experience of HIV patients in Saudi Arabia, healthcare providers and policymakers can better support and enhance the quality of life for this population.

## Introduction

The human immunodeficiency virus (HIV) attacks the immune system, weakening the body’s ability to fight illnesses and infections (Jan et al., 2022). HIV works by impairing the defences that typically guard the body against disease. HIV typically spreads through sexual contact, tainted blood products, and mother-to-child transmission during childbirth or breastfeeding (Yoshimura, 2017). Since its discovery in the early 1980s, HIV has received considerable attention from the medical and scientific communities; despite medical advances, it continues to be a severe and enduring threat to global health (Kleinpeter & Freed, 2020). Although combination antiretroviral therapy (ART) is becoming more widely available and advanced, HIV continues to be blamed for about 1.5 million deaths annually (World Health Organization, 2015). According to the World Health Organization, HIV-related infections caused approximately 630,000 deaths in 2022 (World Health Organization, & HIV, 2023). The global prevalence of HIV cases exhibits significant variation, with African nations, such as Eswatini, reporting the highest rates of HIV incidence (Asangbeh-Kerman et al., 2022). Outside of Africa, Nepal reported the highest level of HIV in Asia (Mahmud et al., 2023). The HIV epidemic continues to worsen in the Middle East and North Africa. An estimated 20,000 new HIV infections occurred in 2019, marking a 25% rise over the 16,000 new cases in 2010 (UNAIDS, 2021). However, in some Middle Eastern countries, the prevalence of HIV remains unclear because of ongoing deficiencies in data collection and reporting (Mumtaz et al., 2022). According to Mumtaz et al. (2022), several countries in the Middle East lack reliable or complete information about HIV trends due to persistent information gaps. In 2018, the Saudi Ministry of Health (MOH) reported an HIV incidence rate of 3 cases per 10,000 individuals in Saudi Arabia (Al-Mozaini et al., 2021). Moreover, in 2022, there were 1300 new cases diagnosed with HIV in Saudi Arabia (UNAIDS, 2022). However, the problems caused by HIV extend beyond the mere number of cases to encompass the everyday difficulties faced by infected individuals living with this disease.

In Saudi Arabia, a public HIV/AIDS awareness campaign conducted by Alwafi et al. (2018) revealed the presence of negative attitudes towards individuals living with HIV/AIDS, potentially stemming from low levels of knowledge. The findings indicated that almost half of the respondents believed that people with HIV/AIDS should be isolated, less than 20% were willing to have a relative marry someone with HIV/AIDS, and nearly 60% expressed reluctance to live with a friend who has HIV/AIDS (Alwafi et al., 2018). In general, the level of HIV/AIDS-related knowledge and attitudes in Saudi Arabia found by Alwafi et al.’s survey is comparable to that found by other studies conducted in the country, most of which reported negative attitudes towards HIV. However, when compared to other countries, knowledge of and attitudes towards HIV/AIDS in Saudi Arabia are relatively poor (Alawad et al., 2019; Kumar et al., 2018).

Individuals with HIV often face stigma and discrimination, particularly within their local communities (Greenwood et al., 2022). Experiencing such stigma and discrimination can cause significant psychological issues for those living with HIV/AIDS, including increased anxiety, depression, and heightened risk of suicidal thoughts or actions (Nasir et al., 2023). Individuals with HIV often live with fear related to family, society, and religion (Parsons, 2022). Fearing the community’s reactions can cause social exclusion and a reluctance to disclose one’s HIV status, exacerbating feelings of loneliness and anxiety (Gabbidon et al., 2020). Moreover, individuals living with HIV often face unique challenges that can contribute to feelings of hopelessness (Nilsson Schönnesson et al., 2022). Thus, It is essential to comprehend their experiences and numerous challenges from their perspective.

In Saudi Arabia, few studies focused on HIV treatment and related adverse impacts have explored the lived experiences of individuals with HIV from the perspective of this group. Gaining insight into the lived experiences of those with HIV is critically important. Their firsthand perspectives show how the virus impacts daily life in a way that statistics alone cannot reveal. Understanding what coping with an HIV diagnosis and managing the illness long-term are genuinely like for individuals helps to inform healthcare providers and health policymakers regarding the disease and highlights barriers to care, unmet needs, sources of stigma, and discrimination experienced. Learning from the challenges and triumphs in the journeys undergone by people living with HIV helps improve treatment outcomes and quality of life. Therefore, this study aimed to understand the lived experience of Saudi patients diagnosed with HIV. The findings of this qualitative study offer valuable insights to healthcare providers, including physicians and nurses, regarding the experiences of this group and reveal the barriers, challenges, and factors that can enhance or hinder their health. By understanding these factors, healthcare providers can improve the quality of care provided to individuals with HIV, ultimately leading to better health outcomes.

## Methods

### Design

The present study utilized hermeneutic phenomenology as it provided the foundation for the individual’s lived experience. Hermeneutic phenomenology aims to uncover phenomena’ underlying essence and significance by engaging in a reflective, interpretive process (Dibley et al., 2020). This approach recognizes that individuals’ experiences are not objective facts but subjective interpretations influenced by their cultural, social, and historical contexts (Dibley et al., 2020). This understanding allows for a deeper exploration of the complexities and nuances within individuals’ experiences, ultimately enhancing the richness and depth of the research findings (Dibley et al., 2020). By utilizing hermeneutic phenomenology, researchers can delve into the intricate layers of meaning and interpretation that individuals assign to their experiences, leading to a more comprehensive understanding of participants’ subjective realities and lived experiences.

According to Heidegger, human beings are self-interpreting entities impacted by ordinary meanings originating from linguistically understandable historical, temporal, and cultural contexts (Heidegger, 1996). Since significance is situated within the particular experience, interpretation can be considered a reflection of explicit comprehension. Due to the complexity of the phenomenon under investigation (having HIV) and the influence of the population who have experienced it (male and female Saudi patients), there arose a necessity for research that examined the lifeworld of the individuals.

### Setting and participants

The researchers used a purposive sample technique to select individuals diagnosed with HIV from a convenience city in the western region of Saudi Arabia. The study’s inclusion criteria were individuals who had been diagnosed with HIV, had lived with the condition for over one year, and had consistently sought medical care for their HIV condition. Individuals afflicted with severe physical or mental illness due to HIV, such as severe depression, were deemed ineligible for participation as it would not have been possible to conduct interviews with them. In qualitative research, the point of data saturation serves as the ultimate criterion for establishing the sample size (Speziale et al., 2011). Data saturation was achieved when 16 interviews had been conducted as no new information emerged.

### Data collection

Mediation was sought from the nurses working at the HIV outpatient clinic to facilitate the engagement of the participants. The nurses assessed the willingness of individuals living with HIV to engage in interviews. To conduct this assessment, the nurses first asked potential participants if they would be interested in speaking with the researcher and then extended an invitation to participate. The researchers presented the participants with an information sheet they could read. If the participants expressed their willingness to participate in the study, they were asked to submit written consent. Participants were encouraged to reflect on their lives before and after being infected with HIV. Interviews were conducted in a separate quiet room prepared previously at the hospital near the HIV outpatient clinic. The participants could terminate the interview at any point if they so desired.

### Interviews

Semi-structured interviews were conducted with individuals living with HIV to obtain comprehensive data on their lived experiences. These interviews were conducted one-on-one, allowing in-depth exploration of participants’ lived experiences. The interviews were conducted entirely in Arabic and lasted between 30 and 45 min. The sessions commenced by initiating an icebreaker question: “Tell me about your lived experience with HIV and how the infection affected your life from your perspective.” This inquiry facilitated the subsequent formulation of more in-depth questions during the interview. The interview guide comprised a set of six primary questions that subsequently prompted further in-depth queries.

### Data analysis

Data analysis of this research was conducted based on Paul Ricoeur, a French philosopher, who argued that a text, such as a transcript, encapsulates the subjective experiences of others (Dreyer & Pedersen, 2009; Ricoeur, 1976). Based on Ricoeur’s inspired method, Dreyer and Pedersen developed a three-fold interpretation approach that results in one or more themes with direct quotes in each theme (Dreyer & Pedersen, 2009). To follow the analysis guide, the audio recordings were transcribed into Arabic by the researchers within 24 hours of the completion of each interview. When all the interviews had been transcribed, each researcher independently translated the transcripts into English. Subsequently, each researcher verified the accuracy of the transcribed translations conducted by their peer. The researchers are bilingual in Arabic and English and hold doctoral degrees from respected academic institutions in the United States. After that, the first fold, the researchers read all transcripts several times. The second fold, the Open and axial coding table, was created to discover relevant themes in the transcripts. Open coding was utilized to analyse each line of the transcripts to identify the most pertinent text and quotation. The open codes were analysed using axial coding to understand the meaning of the extracted text. Finally, in the third fold, a discussion was done by all researchers to find commonalities and distinctions among them and to integrate and create themes.

### Ethical considerations

The research technique adhered to the principles outlined in the Declaration of Helsinki and received approval from the Saudi Ministry of Health Institutional Review Board (IRB log No: 23–015). Informed consent was obtained from all participants, who were explicitly advised of their right to withdraw from the study at any time. Furthermore, measures were taken to arrange the interview location and time to uphold privacy and confidentiality. In order to preserve confidentiality, the researchers did not ask for participants’ names, phone numbers, or addresses. Voice records and transcripts were saved with a password on the researcher’s computer and deleted after the manuscript was written.

### Rigor

The researchers ensured the study’s validity through reflexivity, peer debriefing, and detailed descriptions. The researchers reported this study using the Consolidated Criteria for Reporting Qualitative Research (COREQ) checklist (Tong et al., 2007). In order to attain reflexivity, the research team met once a week to compare and contrast the findings. Another crucial aspect in ensuring validity was the peer debriefing. The researchers invited two infectious diseases medical doctors to engage in reviewing and validating the study’s findings. It is essential to acknowledge that the sensitive nature of the topic and the societal stigma surrounding HIV/AIDS may have limited participants’ willingness to express themselves openly throughout the interviews. However, due to the researchers’ extensive clinical and professional experience, they tried to conduct the interviews in a friendly, non-judgemental way and never asked about how the participants became infected to ensure they felt comfortable and could talk freely. A comprehensive depiction of individuals, motifs, and challenges was ultimately furnished to facilitate readers’ immersion in the narrative.

## Results

Sixteen patients (10 men and 6 women) were interviewed between January 2023 and May 2023. The mean age of participants was 36.6 years (range = 24–52 years). The average duration of living with HIV was 3.7 years (range = 1–9 years). Sixty percent of the participants were married. Six participants had bachelor’s degrees, eight had high school diplomas, and two had graduate degrees. Seven participants were employed, five were unemployed, two were housewives, and two were retired.

Data analysis revealed three main themes and four subthemes: (1) fear of the future with the following subthemes: (a) fear of infecting a family member, (b) fear of marriage, (c) fear of employment recruitment, and (d) fear of scandals); (2) hopelessness; and (3) overcoming adversity.

### Fear of the future

All participants expressed their fear of the future. Fear of infecting a family member, fear of marriage, fear of employment recruitment, and fear of scandals were mentioned by many participants.

### Fear of infecting a family member

Fear of transmitting the disease to a family member was an obsession for all patients. It consumed their thoughts and caused tremendous distress. This fear is not uncommon and can be overwhelming for people living with HIV. Participant 1 stated:

My husband infected me in the second month of my pregnancy. I was sad about my situation and future, but my only fear was that the disease would be transmitted to my child. I used to go to sleep and wake up thinking and praying to God that my innocent child would not be born infected with this disease. The doctors told me and reassured me that by taking medications, there was a great chance that my child would be born safely, but I was not reassured until my child was born, and the doctors told me, after continuous follow-up, that my child was healthy. Then, I felt comfortable.

Some participants thought of getting divorced or separating from their partners out of fear of infecting them. Many participants were vigilant about taking medications consistently and practicing safe behaviours. Unfortunately, the fear of infecting and transmitting the virus to loved ones remains a common concern. Participant 2 said:

I thought about divorcing my wife so as not to transmit the disease to her because she was not at fault for what I did. I know, as doctors told me, that with the regular use of medication and condoms, I can have sexual intercourse with my wife. However, the fear of transmitting the disease to my wife and my love for her always worry me.

### Fear of marriage

The presence of a life partner is a concern for patients with HIV. Some participants are single or divorced, but many want to get married. “I want to get married; I want to live a life with a partner who understands me, and we share a life, but who will accept me! I am afraid nobody will accept me” (Participant 3). Many participants were reluctant to look for a marital relationship due to their apprehension of potential rejection and disappointment. Participant 4 said:

I wish I could get married. Sex is an instinct in the soul of every human being, like eating, drinking, and breathing. I searched for a wife, informed her of my desire to marry, and explained that I have HIV, and she refused to marry me because of the fear of transmitting the disease to her. Because of this experience, I feared being rejected all the time. Ah, I hope to find a wife who is either healthy or HIV-positive. However, I do not expect anyone to accept me.

A participant acknowledged his fear and unwillingness to propose marriage due to expected rejection, “Living life alone is boring. I hope to get married, but I am afraid of rejection, and I expect 90% that I will be rejected because I am HIV-positive” (Participant 5).

### Fear of job-seeking

Medical examinations for jobs constitute an obstacle for patients with HIV applying for posts that require such examinations due to their fear of having their infection discovered and then being rejected by employers. Participant 6, male, expressed his fear in the following words:

I have a college degree and currently work as a taxi driver. Several appropriate jobs are available, but they ask for a medical examination when I apply. Frankly, I am afraid that they will reject me because I am infected.

Some participants preferred to retire from work that required routine medical examination. Participant 7 said:

I was an employee in a prestigious company with a high salary. Still, after I learned I was HIV-positive, I decided to retire early because of the fear of my workplace discovering my status because they conduct annual medical examinations for all employees.

Most participants decided not to apply for any job that requires a medical examination. “I will not apply for any job that requires medical examinations because I do not want to see the looks of contempt and rejection from employers”(Participant 8).

### Fear of scandals

All participants do not want to reveal their medical condition because of the stigma of shame that society directs against people with HIV. Participant 9 said: “My teeth hurt sometimes, but I do not want to go to the dentist because I do not want to tell him that I am HIV-positive, so his view of me changes, and I disgust him.” Many participants experience concern about attending their follow-up visit because their condition may be inadvertently revealed in the presence of others at the outpatient clinics. Participant 10 stated:

Whenever I come to my follow-up appointment at the hospital, I am afraid I will find someone who knows me and sees me in the waiting room or the corridors. I am so scared that they could find out about me. I understand that the hospital deals with me confidentially, and even the clinic where I go to follow up has no sign indicating that it is an HIV clinic, as well as changing its location from time to time to ensure the confidentiality of the patients. However, despite all this, I feel afraid that my condition could be discovered by a colleague or relative who has seen me in the hospital.

Taking sick leave was even an issue with some participants because of the fear of scandal. Participant 11 said:

I sometimes feel tired and would like to take sick leave or send a medical report to my manager, but the clinic’s name, “Infectious Diseases,” is printed on the report. Thus, I am afraid that my manager will read the name of the Infectious Diseases Clinic and that I will start getting looks of pity or contempt from the manager or colleagues.

### Hopelessness

The hopes and dreams of the participants changed after they were infected. Participant 12 stated:

I dreamed of continuing my postgraduate studies, getting married, and having the house and car of my dreams. Now, after the disease, my only dream is that God forgives my sin and that I can live the rest of my life in psychological and physical peace and not die because of the complications of HIV.

Some Participants had no personal goals or hopes they would achieve in the future. They want to live to raise their children. “My only hope is my children, and they are the only reason for my survival. I hope to see them in the best position, but on a personal level, HIV has removed all my future dreams” (Participant 13). Many participants had similar feelings, “ … .I live my days only for my child” Participant 14. Hopelessness was a familiar feeling among all the participants, “Ahh, what dreams are you talking about?…. Since I learned that I was HIV-positive, I have been counting the days remaining in my life” (Participant 15). All participants’ hopes and dreams shifted towards HIV complications and negative consequences. Participant 16 stated: “My dreams had no limits. After being infected, I became socially isolated, and all my focus was on the percentage of the virus. My dreams have gone.”

### Overcoming adversity

Almost all the participants who overcame the bad news of being HIV patients mentioned that they had massive support from their family or friends and had returned to religion and the idea of predestination. Participant 2 stated: “When I learned the test result, I could not bear the horror of the news and could not accept it, but my brother supported me and reminded me of God and helped me overcome this ordeal.”

Partners’ support was essential to overcoming HIV condition, Participant 7 said:

My wife, who I expected would ask for a divorce from me after I had HIV, surprised me with her support, and she was the best help for me to live with this disease and adhere to treatment. She always told me that HIV is a chronic disease, like high blood pressure and diabetes, and by taking medications, you can live.

Moreover, many participants found that believing in God’s predestination and what God has chosen for them played a role in accepting their condition; “Returning to God and remembering that getting HIV was by God’s command helped me a lot” (Participant 9).

## Discussion

A qualitative study was conducted to understand the lived experience of patients with HIV. The findings of this research offer valuable insights for healthcare providers, including physicians and nurses, shedding light on the distinctive perspectives and encounters of individuals living with HIV. The outcomes of this study can contribute substantial knowledge to inform and guide healthcare professionals in delivering tailored care and support to meet better the needs of people living with HIV. This qualitative phenomenology study revealed several themes from the interviews with HIV patients.

### Fear of the future

Fear of the future was the first theme observed after the data were analysed and represents a significant emotional burden experienced by many of our participants. The uncertain nature of the disease, potential health complications, and long-term implications of HIV can create anxiety and apprehension about what lies ahead (Kimera et al., 2020). Fear may stem from concerns about disease progression, the effectiveness of treatment options, and the impact on one’s personal and professional life. The participants in our study consistently conveyed a prevalent fear of their future, stemming from a sense that their lives had come to a halt. This fear was deeply rooted in the profound impact of living with HIV, which often led to a disruption of their life trajectories and a re-evaluation of their goals and aspirations. Our participants expressed concerns about the potential limitations imposed by their HIV status, including the impact on their careers, relationships, and overall quality of life, similar to the findings of the phenomenology study conducted by Asadi et al. (2018).

The participants in our study placed significant emphasis on the fear of being responsible for transmitting HIV to other loved ones; this finding is similar to that of other studies in the literature in which patients express their concerns that they might be responsible for transmitting HIV to others, especially their family (Evangeli & Wroe, 2017; Sineke et al., 2023). In particular, patients expressed that fear of transmitting the disease to their children or spouse was a concern. This fear reflected their deep sense of responsibility and love for their family members. Participants expressed anxiety and worry about unintentionally infecting their children or spouse with HIV, which intensified their fear of the future (Appiah et al., 2019). According to Fauk et al. (2023), parents diagnosed with HIV often have a profound concern about being the source of infection for their children, who may be living with the same disease. However, during hospital visits, clinicians play a crucial role in providing clinical support and imparting instructions to enhance patients’ understanding of the disease transition methods and enable them to lead everyday lives with their families (Belyeu et al., 2018).

Single or divorced patients living with HIV often express concerns about the prospect of getting married in the future (Sastre et al., 2015). In Saudi Arabia, where religious and cultural norms encourage marriage and having children, these norms increase the pressure on HIV patients. However, stigma against HIV patients contributes to the refusal of other members of society to marry them (Zhang et al., 2016). This stigma perpetuates the notion that individuals with HIV are somehow “undesirable” as potential partners, causing them to face additional emotional and psychological challenges in their pursuit of love and companionship (Omer et al., 2014). The presence of stigma towards individuals living with HIV is a consistent finding across multiple phenomenological studies (Imani et al., 2021; Jahromy et al., 2021; Sadati et al., 2019).

In Saudi Arabia, the Saudi MOH has implemented a programme to assist single or divorced individuals diagnosed with HIV to find suitable marriage partners living with the same condition. The insights shared by the participants and healthcare providers regarding the support received by HIV patients in Saudi Arabia are valuable in highlighting the efforts and support systems in place within the country.

In our study, patients with HIV expressed concerns about pursuing a career and maintaining employment. The fear of discrimination, stigmatization, and potential disclosure of their HIV status can create barriers and anxieties around finding and retaining employment (Alharbi et al., 2022). The finding of our study is consistent with the finding study conducted by Imani et al. (2021), where they reported that discrimination against individuals living with HIV is a distressing reality that is often documented in phenomenological studies. These concerns may compromise the desire to lead fulfilling and productive lives, contribute to society, and secure financial stability. It is crucial to address these apprehensions and provide support to HIV patients in navigating the workplace. Such support can involve educating employers and colleagues about HIV, promoting inclusivity and non-discrimination policies, and ensuring access to reasonable workplace accommodation and healthcare benefits. By addressing these concerns and providing a supportive work environment, individuals living with HIV can pursue their career goals and thrive professionally while managing their health effectively. It is worth noting that the Saudi government launched a system for preventing acquired immunodeficiency syndrome (AIDS) and the rights and duties of those infected in 2018. This system clarifies the rights and duties of HIV patients (Bureau of Experts at the Council of Ministers, 2018).

Fear of scandal related to HIV is a significant concern in Arab countries (Alwafi et al., 2018). During the interview with study participants, most of them considered HIV as a scandal. This might be influenced by the cultural perspective towards individuals diagnosed with HIV. The strong influence of cultural and societal norms, combined with the stigma surrounding HIV, can create apprehension and anxiety among individuals living with the virus (Badahdah, 2010). The fear of public exposure, judgement, and potential repercussions on personal and familial reputation can be overwhelming (Imani et al., 2021; Tran et al., 2019). These concerns may deter individuals from seeking testing, treatment, and support services, leading to delayed diagnosis and limited access to the necessary care. According to Kiwanuka et al. (2022), lack of support, fear of stigma, and exposure were among the themes expressed by their study participants, which contributed to low health-seeking behaviour among HIV patients. It is crucial to address and combat the stigma associated with HIV in Arab countries through awareness campaigns, education, and advocacy (Alwafi et al., 2018). By promoting a more compassionate and understanding society, individuals with HIV can feel empowered to seek help, access appropriate care, and live fulfilling lives without the constant fear of scandal.

### Hopelessness

Hopelessness was expressed by most of our participants, as many of them had lost hope and felt they would not achieve their dreams. According to Nilsson Schönnesson et al. (2022), hopelessness is a common emotional experience among HIV patients. In our study, participants expressed hopelessness, as many of them indicated that there is no final cure for the disease. Hope to cure is one of the factors that influence HIV patients’ health-seeking behaviour and was found to be a critical theme in a qualitative study conducted by Mohammadpour et al. (2014). The chronic nature of the disease, potential health complications, and the societal stigma associated with HIV can contribute to a profound sense of despair (Deshmukh et al., 2017).

Moreover, the uncertainty surrounding the future, including concerns about disease progression, treatment efficacy, and social isolation, can exacerbate hopelessness. The challenges posed by HIV can make individuals feel trapped and overwhelmed, leading to a loss of optimism and motivation (Cavazos-Rehg et al., 2021). Healthcare providers, support groups, and mental health professionals must address and alleviate these feelings of hopelessness through comprehensive care, counselling, and psychosocial support. Suppose a sense of hope and resilience is fostered. In that case, patients can regain a positive outlook and find the strength to overcome the challenges of living with HIV, leading to improved overall well-being and quality of life.

### Overcoming adversity

Overcoming adversity was the last theme that emerged from our study, as all participants stated they had at least one person to support them during this challenging time, whether from siblings or wives. Family support plays a crucial role in the lives of patients diagnosed with HIV (Xu et al., 2017). Family members’ understanding, love, and acceptance can significantly impact the well-being and quality of life of individuals living with the virus (Xu et al., 2017). In our study, we noticed that most participants had one family member who knew about their situation. Most do not want anyone else to know except one who could understand and support them. The support received from loved ones, either participants’ siblings, if they are single, or their spouse, if they are married, was necessary and contributed to better adherence to treatment regimens and improved health outcomes. Moreover, regarding the importance of religiosity in coping with HIV, Brito and Seidl (2019) found, similarly to our study, that religious coping can influence people’s abilities to overcome the adversities related to living with HIV and should, therefore, be routinely evaluated by healthcare providers.

According to the findings of our study, patients’ most significant apprehension revolved around the ability to lead an everyday life, including getting married, pursuing a career, and building a future for themselves. This fear can be overwhelming, leading to feelings of hopelessness and a sense of being trapped by the disease. According to the abovementioned discussion, some of these concerns are similar among patients with HIV around the world. However, cultural sensitivity is paramount when addressing HIV in Saudi and Arab cultures (Alageel & Alomair, 2023). Culturally sensitive approaches involve considering and understanding the cultural factors influencing attitudes, beliefs, and HIV-related behaviours (Moradi et al., 2016), including religious teachings, family dynamics, gender roles, and societal expectations (Fauk et al., 2021). Healthcare providers and educators must know these cultural nuances and adapt their approaches accordingly.

### Recommendations for healthcare policymakers and future researchers

Based on the researchers’ field observations, some HIV clinics in Saudi Arabia need to adopt a multidisciplinary care team to provide holistic care for HIV patients. This might improve patients’ health outcomes and enhance the quality of care. Future researchers may apply and measure the effects of a multidisciplinary care team on HIV patients’ satisfaction rates and health outcomes. HIV patients face many challenges in their lives. Fear of the future, including fear of infecting a family member, fear of marriage, fear of employment recruitment, and fear of scandals, are daily concerns that negatively affect patients’ lives. Interventions such as support groups, treatment by peers, or psychiatric counsellors must be developed to mitigate hopelessness and help patients with HIV overcome adversity.

### Limitations

This study has two limitations. First, we only interviewed HIV patients in an HIV outpatient clinic. The perspectives of those patients not registered with and attending the clinic are unknown. Second, this study was conducted at one centre. Therefore, multi-centres in different cities are needed.

## Conclusion

The experience of living with HIV is a complex and multifaceted journey that encompasses emotional and social challenges. The fear of the future, including concerns such as infecting family members, marriage prospects, employment opportunities, and potential social repercussions, significantly impacts their overall well-being. Healthcare professionals must understand and comprehend the complexity of living with HIV and the patients’ existential needs to provide tailored interventions for patients with HIV.

## Data Availability

The data that support the findings of this study are available from the corresponding author upon reasonable request.
